# Prophylactic Swallowing Therapy During Head-and-Neck Cancer Radiotherapy: Effect of Service-Delivery Mode and Overall Adherence Level on Swallowing Function and Muscle Strength—the PRESTO Trial

**DOI:** 10.1007/s00455-023-10609-7

**Published:** 2023-08-08

**Authors:** Margot Baudelet, Leen Van den Steen, Fréderic Duprez, Ann Goeleven, Sandra Nuyts, Daan Nevens, Caroline Vandenbruaene, Hanne Massonet, Alice Vergauwen, Tom Vauterin, Hilde Verstraete, Kristien Wouters, Olivier Vanderveken, Marc De Bodt, Gwen Van Nuffelen, members of the Belgian PRESTO-group

**Affiliations:** 1 Department of Radiation Oncology, University Hospital Ghent, 9000 Ghent, Belgium; 2 Department of ENT, University Hospital Ghent, 9000 Ghent, Belgium; 3 Faculty of Medicine and Health Sciences, University of Antwerp, Antwerp, Belgium; 4 University Hospital Antwerp, Antwerp, Belgium; 5 Faculty of Medicine and Health Sciences Ghent, University of Ghent, Ghent, Belgium; 6 Department of Head and Neck Surgery, Swallowing Clinic, University Hospital Leuven, Louvain, Belgium; 7 Faculty of Medicine, Department of Neurosciences, Research Group Experimental Oto-rhino-laryngology, KU Leuven, Louvain, Belgium; 8 Laboratory of Experimental Radiotherapy, Department of Oncology, KU Leuven, 3000 Leuven, Belgium; 9 Department of Radiation Oncology, Leuven Cancer Institute, University Hospitals Leuven, 3000 Leuven, Belgium; 10 Multi-Disciplinary Oncology Center Antwerp, Antwerp, Belgium; 11 Iridium Network, Antwerp, Belgium; 12 AZ Sint-Jan Brugge, Brugges, Belgium; 13 Clinical Trial Center (CTC), CRC Antwerp, Antwerp University Hospital, University of Antwerp, Edegem, Belgium

**Keywords:** Dysphagia, Deglutition, Deglutition disorders, Head-and-neck cancer, Adherence, Prophylactic swallowing exercises, (Chemo) radiotherapy, Swallowing function, Muscle strength

## Abstract

Prophylactic swallowing exercises (PSE) during head-and-neck cancer (HNC) (chemo)radiotherapy (CRT) have a positive effect on swallowing function and muscle strength. Adherence rates to PSE are, however, moderate to low, undermining these effects. PRESTO already showed that the service-delivery mode (SDM), the way the exercises are offered, can influence adherence. The aim of this study was to investigate the effect of SDM on swallowing function and muscle strength during and post-CRT. In addition, the effect of overall adherence (OA), independent of SDM, was also investigated. A total of 148 HNC patients, treated with CRT, were randomly assigned to one of the three SDM’s (paper-supported, app-supported, or therapist-supported PSE) and performed a 4-week PSE program. OA was calculated based on the percentage of completed exercises. Patients were divided into OA levels: the OA75+ and OA75− group performed respectively ≥ 75 and < 75% of the exercises. Swallowing function based on Mann Assessment of Swallowing Ability-Cancer (MASA-C), tongue and suprahyoid muscle strength during and up to 3 months after CRT were compared between the SDM’s and OA levels. Linear Mixed-effects Models with post hoc pairwise testing and Bonferroni–Holm correction was used. No significant differences were found between the three SDMs. Significant time effects were found: MASA-C scores decreased and muscle strength increased significantly during CRT. By the end of CRT, the OA75+ showed significantly better swallowing function compared to OA75−. Muscle strength gain was significantly higher in the OA75+ group. SDM had no impact on swallowing function and muscle strength; however, significant effects were shown for OA level. Performing a high level of exercise repetitions is essential to benefit from PSE.

*Trial registration* ISRCTN, ISRCTN98243550. Registered December 21, 2018—retrospectively registered, https://www.isrctn.com/ISRCTN98243550?q=gwen%20van%20nuffelen&filters=&sort=&offset=1&totalResults=2&page=1&pageSize=10&searchType=basic-search

## Background

During the last decade, the use of prophylactic swallowing exercises (PSE) in patients treated with radiotherapy or concomitant chemoradiotherapy (RT/CRT) for head-and-neck cancer (HNC) is gaining more interest [1–4]. The rationale behind these prophylactic strategies is prevention of weakness and disuse atrophy of the swallowing musculature [4, 5]. Previous research showed that prophylactic swallowing therapy can lead to less muscle atrophy and an improved dysphagia-related QoL with less aspiration, less feeding tube dependency and less hospitalization post-treatment [1, 2, 6, 7]. Adherence rates to PSE are, however, moderate to low (71–13%) and typically decline during RT/CRT [8–12]. This threatens the positive effect the exercises have. Duarte et al. showed that the swallowing function in patients who were adherent to PSE exercises was better preserved at the end of RT/CRT than in patients who were not adherent to the exercises [6]. Moreover, Peng et al. observed no significant differences between pre- and post-treatment swallowing function in patients who adhered to the PSE exercises, whereas patients who did not adhere to them showed a tendency toward worse swallowing function [13].

Previous research indicated already that the way the exercises are given, the service-delivery mode, has a significant effect on patients’ adherence [14, 15]. Most commonly reported service-delivery modes for PSE are diary-supported home practice, app-supported home practice, and speech-language pathologist (SLP)-supported practice [2, 3, 14, 16, 17]. Wall and colleagues compared adherence rates in those three groups and found during week 1–3 of RT/CRT significant higher rates in patients performing SLP-supported PSE compared to patients practicing at home, without supervision. However, in general, adherence rates were low (27%) during the 6 training weeks in all groups, although there was a trend towards higher rates in the app-supported group compared to the home practice group [14]. PRESTO also investigated the effect of three different service-delivery modes on the actual adherence to PSE and demonstrated significant differences in adherence between the three modes with highest rates in the group practicing face to face with the SLP, followed by high to medium rates in the group practicing at home with a diary. Patients practicing at home with the help of an online application had moderate to low adherence rates [18]. The question arises whether service-delivery mode of PSE can also impact on swallowing function and muscle strength during and post-RT/CRT. This was based on following findings:

1. Previous research showed that adherence needs to be high enough to show effects on swallowing function [6, 13].
2. PRESTO showed that service-delivery mode has an impact on adherence [18].
3. PRESTO showed how to keep adherence rates high [18].

The aim of this study was to investigate the effect of the three different service-delivery modes for executing an intensive PSE program on the swallowing function and muscle strength in HNC patients. In addition, the effect of overall adherence (OA), based on the total percentage of completed exercises, was assessed.

## Methods

### Study Design and Participants

The Prophylactic Swallowing Exercise Therapy program for patients with Oropharyngeal cancer (PRESTO) trial is a multicenter, prospective randomized controlled trial (RCT). Patients with stage III or IVA-B (TNM7) newly diagnosed squamous cell carcinoma of the oropharynx were recruited at four Belgian  hospitals (University Hospitals of Antwerp/Iridium Network, Ghent and Leuven and General Hospital Sint-Jan Bruges). Potential candidates were both men and women, > 18 years old, showing no cognitive or language deficits. Patients were treated with 6–7 weeks fractionated RT/CRT with or without induction chemotherapy. Exclusion criteria were the presence of a recurrent carcinoma or metastasis from a non-HNC carcinoma and previous RT/CRT or surgery in the head–neck region with possible impact on swallowing function.

All subjects who gave written informed consent to participate in the study, were randomly assigned to one of the following service-delivery modes: paper-supported prophylactic swallowing exercises (PSE) (paper group), app-supported PSE (app group), or therapist-supported PSE (therapist group), and this by means of the minimization program QMinim.

All participants, irrespective of their assigned group, performed a 4-week PSE program for 5 days a week. Since acute toxicity becomes excessively pronounced from the fifth week of RT/CRT, affecting patients’ adherence, the duration of the program was limited to the first 4 weeks of RT/CRT [8, 15], whereas previous studies applied PSE during the complete RT period [2, 13]. The PSE program consisted of two evidence-based exercises, alternating daily and targeting the main muscle groups involved in swallowing. First, tongue-strengthening exercises were performed by using the Iowa Oral Performance Instrument (IOPI, model 3.2, IOPI Medical LLC, Woodinville, WA, USA) and consisted of 120 tongue presses per session, divided into twelve sets of ten repetitions. Second, chin tuck against resistance exercises was done by using the Swallowing Exercise Aid [19] and one session consisted of 150 chin tucks, divided into 30 sets of five repetitions. The fifth repetition was a combination of a chin tuck with an effortful swallow. For both exercises, patients were asked to complete the full set of repetitions. Patients practiced at 60–80% of their 1repetition maximum (1RM), depending on the exercise. The 1RM was the highest value out of three trials, which was remeasured and recalculated every week by means of the IOPI Pro and a dynamometer [20, 21].

### Service-Delivery Mode

The three service-delivery mode groups differed in degree and kind of adherence-improving measures. The first group, the paper group, received a logbook and written instructions to practice at home. They were asked to register how many exercises they performed and if they experienced any difficulties. The second group, the app group, practiced at home using an application, which included instructional videos for the patients to re-watch as many times as needed. Repeated instructions were given through the app and gamification was used to make the difficult task more pleasant. The patients registered via the app how many exercise repetitions they did and if any difficulties arose. More detailed information on the development and content of the application was published previously [18]. In both groups, the first session was completed under supervision of the SLP and every week an appointment was scheduled to recalculate the target value. The third group, the therapist group, was given face-to-face therapy for 5 days/week. Each session, clear and repeated instructions were given and patients received continuous feedback on their performance. The SLP kept a logbook and registered how many exercises the patients did.

### Primary Outcome: Swallowing Function

The primary outcome of this RCT was the swallowing function, based on the Mann Assessment of Swallowing Ability-Cancer (MASA-C) [22]. The MASA-C is a reliable and valid swallowing assessment tool that is sensitive to detect differences in swallowing performance in HNC patients with and without dysphagia. In this study, it was conducted with three different bolus types: 10 ml of thin liquid (IDDSI [international dysphagia diet standardization initiative [23]] 0), 10 ml of thickened water (IDDSI 3), and one bite of a cake (IDDSI 6). The maximum score is 200, referring to normal swallowing, scores beneath 186 refer to dysphagia. When patients refused to eat the thickened water or cake due to any reason, and thus, making it impossible to correctly evaluate their swallowing act, the lowest score on this subtest of items (swallowing act) was given.

### Secondary Outcome: Muscle Strength

Tongue and suprahyoid muscles strength were secondary outcome measures. The IOPI Pro, model 3.1 (IOPI Medical LLC, Woodinville, WA, USA) was used to measure maximal anterior and posterior tongue strength and tongue strength during a dry swallow. The location to measure anterior maximal isometric pressure (MIP_a_) was determined by placing the proximal end of the bulb immediately behind the upper teeth at the midline of the palate. The location for the posterior MIP (MIP_p_) was defined by placing the main part of the bulb at the level of the transition from the hard to the soft palate. Patients are asked to push the bulb as hard as possible against the palate while the exerted pressure in kilopascal (kPa) is shown on the LCD screen of the IOPI. The highest value of three trials was considered the MIP. To obtain the tongue strength during swallowing (Pswal), participants were asked to execute an effortful saliva swallow with the tongue bulb in the same positions as MIP_a_ and MIP_p_ for respectively Pswal_a_ and Pswal_p_. Again, the highest value of three trials was considered to be the Pswal and used for analysis. The maximal strength of the suprahyoid muscles (MIP_shm_) was measured by means of a dynamometer (Microfet™, Biometrics, Almere, The Netherlands) [24]. Participants were asked to place their chin on the chin bar, keep their mouth and teeth closed, and press their chin down as hard as possible while the patients’ head is stabilized by a fixed belt. The exerted pressure is shown in Newton (N), and the highest value of three trials is considered the maximal isometric chin-tuck strength.

The evaluation of swallowing function based on MASA-C as well as the measurements of muscle strength were done by the SLP at baseline, every week during the 4 weeks of PSE, at the end of RT/CRT, and 1 and 3 months after RT/CRT. When a patient was treated with induction chemotherapy, the baseline measurement was performed immediately before the start of radiotherapy.

The full protocol has been described and published previously [21].

### Overall Adherence

As an additional analysis, the effect of overall adherence (OA), irrespective of service-delivery mode, on swallowing function and muscle strength was also investigated. Participant’s OA was computed by summing all repetitions during the 4 training weeks, dividing this by the maximum number of repetitions (i.e., 2700 reps) and multiplying it by 100. Based on their OA, the PRESTO-participants were regrouped in four OA levels: OA75+ , performing ≥ 75% of the prescribed exercises, i.e., high practice, OA50-75, performing 50–75% of the prescribed repetitions, i.e., moderate practice, OA25-50, performing 25–50% of the exercises, i.e., low practice, and OA25−, performing < 25% of the exercises, i.e., negligible practice [14].

### Statistical Analysis

#### Sample Size Calculation

The sample size calculation was performed using GLIMMPSE online software for power calculation in linear mixed effects models. The targeted total sample size, taking into account 20% dropouts, was 150 (*n* = 50/group, depending on minimization). More details on sample size calculation are presented in the protocol publication [21].

#### Data Analysis

Descriptive statistics were used to summarize patient characteristics per service-delivery mode group and per OA level.

For MASA-C, a linear mixed effects model with group, time and group by time interaction as fixed effects was used. In addition, the same model was corrected for OA level as fixed effect. The interaction effect of group by time was removed out of the model when no significant results were observed. The final model included group, time, adherence level, and adherence level by time interaction as fixed effects. In case significant time effects were found, post hoc pairwise testing with Bonferroni–Holm correction for multiple testing was performed for the results between baseline and the end of RT/CRT, baseline, and 3 months post-RT/CRT and between end of RT/CRT and 3 months post-RT/CRT. When significant group/OA level effects were found, post hoc pairwise testing with Bonferroni–Holm correction for multiple testing was performed for the results at baseline, end of RT/CRT, and 3 months post-RT/CRT.

For muscle strength, the percentage of strength gain or loss compared to baseline was systematically calculated. A linear mixed effects model with group, time, and group by time interaction as fixed effects was used. Again, in addition, the model was corrected for OA level as fixed effect, and when no significant group by time interaction effects was found, this was removed from the model. The final model included group, time, adherence level, and adherence level by time interaction as fixed effects. In case, significant time effects were found, post hoc pairwise testing with Bonferroni–Holm correction for multiple testing was performed for the results between baseline and week 1 of RT/CRT, baseline and the end of RT/CRT, baseline and 3 months post-RT/CRT, and between end of RT/CRT and 3 months post-RT/CRT. When significant group/OA level effects were found, post hoc pairwise testing with Bonferroni–Holm correction for multiple testing was performed for the results at the end of RT/CRT and 3 months post-RT/CRT.

We hypothesized better swallowing function and improved muscle strength in (1) patients in the therapist group when comparing the 3 service-delivery modes and (2) patients in the OA75+ group compared to the other OA levels.

Data were assumed to be missing at random. In the linear mixed effects model, all information on the available time points is incorporated. Since for MASA-C IDDSI 0 only 11.6% of data were missing, we did not perform a sensitivity analysis using multiple imputation, as described in our statistical analysis plan.

Patients who dropped out during the PSE weeks due to medical circumstances or patients who lost (parts of) there logbook, were not included in the OA analyses. The number of exercises performed during that/those specific week(s) must be considered as missing instead of zero. These missing values made it impossible to assign these participants to an OA level. Since > 20% of the patients had missing values for adherence, a sensitivity analysis was performed. In this sensitivity analysis, patients with missing data were assigned into an OA level based on the available adherence data and the knowledge that the adherence rates will not increase over time during RT/CRT [8, 9, 18].

A *p* value of < 0.05 was considered statistically significant. All analyses were conducted using SPSS Statistics version 27 (IBM, Chicago, IL, USA).

## Results

### Participants

**Fig. 1:**
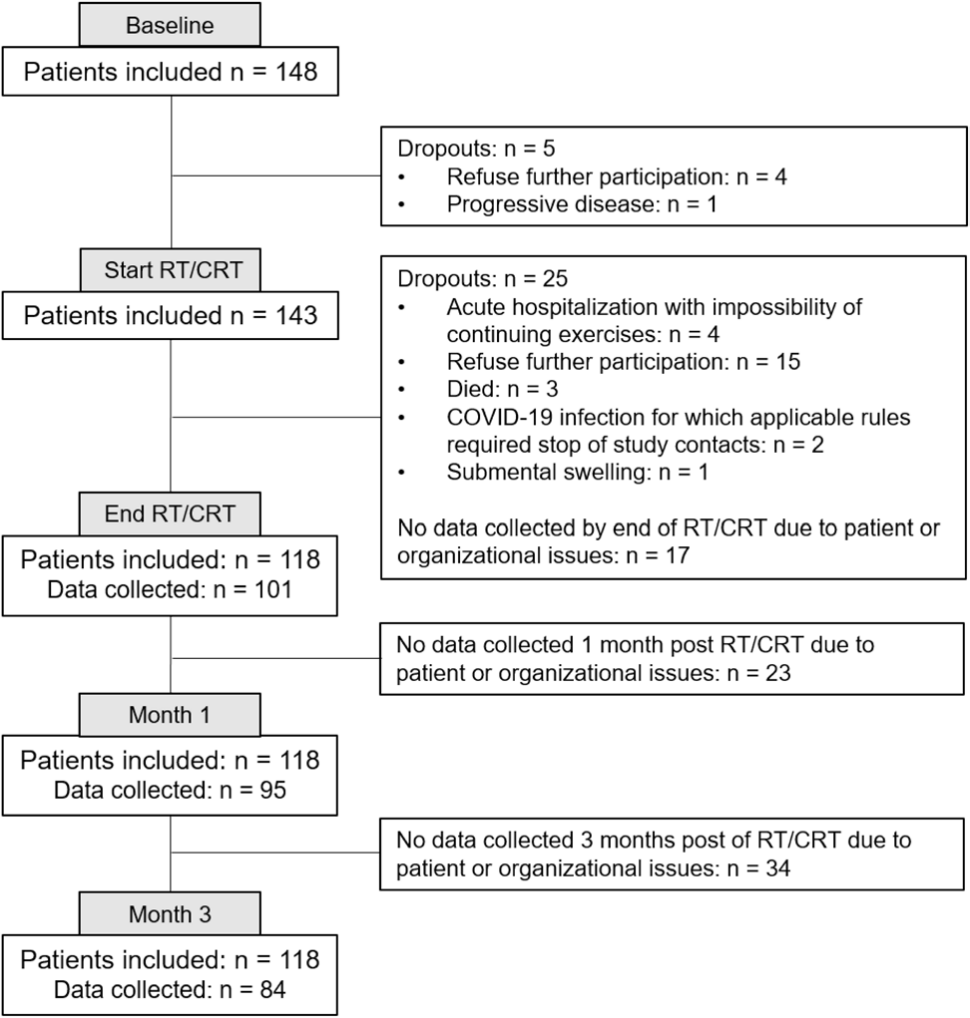
Flowchart patient inclusion, dropouts, and follow-up

One hundred and fifty patients were recruited for this study. Two patients were excluded from this cohort. The first patient was excluded due to a change in the study protocol, namely by adding the exclusion criteria of having a tracheotomy influencing the execution of the CTAR exercise. The second patient was hospitalized due to an acute life-threatening disease before baseline measures were conducted and could therefore not participate in the study. Finally, a cohort of 148 patients was maintained for further analysis. Patient, disease, and treatment characteristics of the whole cohort and separate service-delivery mode groups can be found in Table 1. Figure 1 shows a flowchart of the patients’ inclusion, dropouts and follow-up.

**Table 1:** Patient, disease and treatment characteristics of both SDM groups and OA levels, and number of patients per OA level by service-delivery mode

	Total cohort N = 148 (%)	Paper group N = 49 (%)	App group N = 49 (%)	Therapist group N = 50 (%)	OA75+ N = 62 (%)	OA75− N = 53 (%)
Age	M = 63 SD = 8.5 Range = 41–86	M = 63 SD = 9.5 Range = 41–86	M = 63 SD = 7.9 Range = 41–83	M = 63 SD = 8.2 Range = 45–80	M = 64 SD = 8.6 Range = 41–80	M = 62 SD = 8.0 Range = 50–83
Gender						
Female	35 (24)	14 (29)	11 (22)	10 (20)	9 (15)	17 (32)
Male	113 (76)	35 (71)	38 (78)	40 (80)	53 (85)	36 (68)
T classification						
1	25 (17)	9 (18)	7 (14)	9 (18)	13 (21)	6 (11)
2	51 (34)	17 (35)	15 (31)	19 (38)	20 (32)	21 (40)
3	39 (27)	13 (27)	12 (25)	14 (28)	18 (29)	13 (24)
4	12 (8)	3 (6)	5 (10)	4 (8)	4 (6)	4 (8)
4a	18 (12)	7 (14)	8 (16)	3 (6)	6 (10)	9 (17)
4b	3 (2)	0 (0)	2 (4)	1 (2)	1 (2)	0 (0)
N classification						
0	7 (5)	3 (6)	3 (6)	1 (2)	1 (2)	4 (7)
1	23 (16)	7 (14)	7 (14)	9 (18)	11 (18)	5 (9)
2	5 (3)	1 (2)	2 (4)	2 (4)	2 (3)	3 (6)
2a	10 (7)	5 (10)	1 (2)	4 (8)	7 (11)	1 (2)
2b	54 (36)	17 (35)	20 (41)	17 (34)	19 (30)	20 (38)
2c	40 (27)	14 (29)	12 (25)	14 (28)	19 (30)	16 (30)
3	9 (6)	2 (4)	4 (8)	3 (6)	3 (5)	4 (8)
Treatment						
RT	21 (14)	6 (12)	8 (16)	7 (14)	10 (16)	6 (11)
CRT	102 (69)	37 (76)	32 (65)	33 (66)	40 (65)	40 (76)
CRT + induction CT	25 (17)	6 (12)	9 (19)	10 (20)	12 (19)	7 (13)
HPV status						
Positive	76 (51)	24 (49)	23 (47)	29 (58)	35 (56)	27 (51)
Negative	72 (49)	25 (51)	26 (53)	21 (42)	27 (44)	26 (49)
Dysphagia at baseline, based on MASA-C						
No	119 (80)	39 (80)	38 (78)	42 (84)	49 (79)	43 (81)
Yes	29 (20)	10 (20)	11 (22)	8 (16)	13 (21)	10 (19)
Treating center						
UZ Antwerpen/Iridium Network	32 (22)	11 (22)	10 (20)	11 (22)	11 (18)	11 (21)
UZ Gent	57 (38)	19 (39)	19 (39)	19 (38)	26 (42)	23 (43)
UZ Leuven	41 (28)	12 (25)	14 (29)	15 (30)	16 (26)	12 (23)
AZ Sint-Jan Brugge	18 (12)	7 (14)	6 (12)	5 (10)	9 (14)	7 (13)

	Total cohort N = 115 (%)	Paper group N = 36 (%)	App group N = 34 (%)	Therapist group N = 45 (%)
OA level				
OA25−	13 (11)	3 (8)	10 (29)	0
OA25-50	9 (8)	4 (11)	4 (12)	1 (2)
OA50-75	31 (27)	8 (22)	12 (35)	11 (24)
OA75+	62 (54)	21 (58)	8 (24)	33 (73)
OA75−	53 (46)	15 (42)	26 (77)	12 (27)
OA75+	62 (54)	21 (58)	8 (23)	33 (73)

There were 26 dropouts before or during prophylactic swallowing exercises (PSE) (i.e., before or during the first 4 weeks of RT/CRT), exercise data on tablets were not correctly saved in four patients and another three patients lost their exercise logbook. This resulted in these 33 patients not being able to be assigned into an overall adherence (OA) level, leading to the inclusion of 115/148 (78%) patients into these different categories.

Since the number of patients per OA level was too small to allow for comparison between the four groups, the choice was made to combine groups: OA75+ vs. OA75− (consisting of OA25−, OA25-50 and OA50-75). Additionally, reducing the number of OA levels as described, resulted in a better model fit and Akaike’s Information Criteria (AIC) compared to the models for the four separate OA levels.

Table 1 shows the number of patients per OA level by service-delivery mode and the patient, disease, and treatment characteristics of the OA75+ and OA75−.

### Swallowing Function Based on MASA-C

**Fig. 2:**
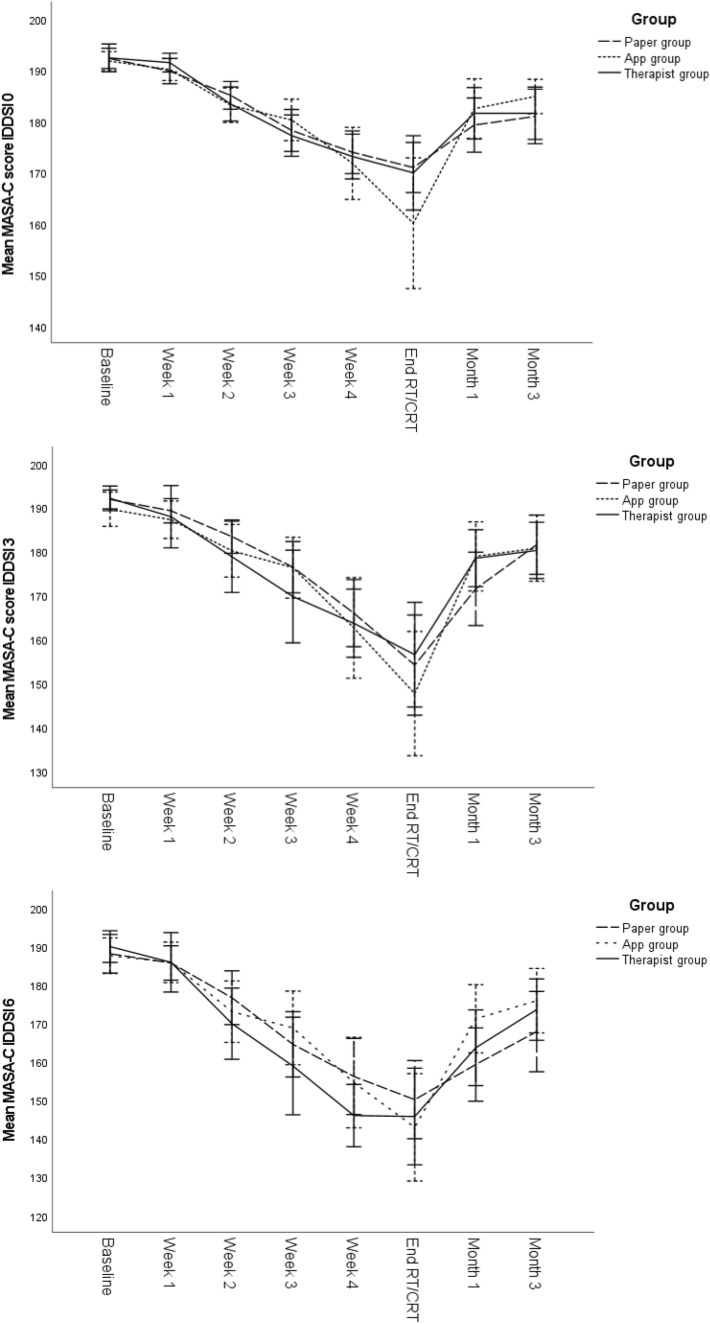
Evolution of MASA-C IDDSI 0, 3, and 6 scores over time, error bars 95% CI

Figure 2 shows the evolution of MASA-C IDDSI 0, 3, and 6 scores over time per adherence group.

Linear mixed effects model with time, group, and group by time interaction shows a significant interaction effect on IDDSI 0 (*F*_14–695_ = 1.859, *p* = 0.028). When correcting this model for adherence, with adherence as a categorical variable (OA75+ vs. OA75−), this interaction effect is no longer observed, from which we infer that adherence, rather than group, affects swallowing function. Since group by time interaction is no longer significant, this variable was removed in our model.

In the linear mixed effects model with group, time, adherence, and adherence by time interaction, significant time effects are observed for IDDSI 0 (*F*_7–637_ = 88.187, *p* < 0.001), IDDSI 3 (*F*_7–584_ = 56.368, *p* < 0.001) and IDDSI 6 (*F*_7–567_ = 71.811, *p* < 0.001), significant effects of adherence are also observed for IDDSI 0 (*F*_1–112_ = 5.395, *p* = 0.022), IDDSI 3 (*F*_1–112_ = 7.566, *p* = 0.007), and IDDSI 6 (*F*_1–110_ = 4.215, *p* = 0.042). Adherence by time interaction is significant for IDDSI 0 (*F*_7–637_ = 2.171, *p* = 0.035) and IDDSI 3 (*F*_7–584_ = 2.875, *p* = 0.006), however, not for IDDSI 6 (*F*_7–567_ = 1.237, *p* = 0.280).

Post hoc analyses with Bonferroni–Holm correction show significant decreases in MASA-C scores between baseline and the end of RT/CRT and between baseline and 3 months post-RT/CRT. Significant increases between the end of RT/CRT and 3 months post-RT/CRT were observed. These results apply for all three consistencies and both groups. Results are shown in Table 2.

**Table 2:** Results of post hoc tests with Bonferroni–Holm correction for the evolution of MASA-C scores through time, depending on group

		MASA-C IDDSI 0		MASA-C IDDSI 3		MASA-C IDDSI 6	
Difference in time-point							
		Estimate* (95% CI)	*p*	Estimate (95% CI)	*p*	Estimate (95% CI)	*p*
OA75+							
Baseline	End RT/CRT	− 21.11 [− 24.33 to − 17.89]	*< .001*	− 32.19 [− 38.49 to − 25.89]	*< .001*	− 40.81 [− 47.70 to − 33.91]	* < .001*
Baseline	3 months	− 11.74 [− 15.16 to − 8.33]	*< .001*	− 11.32 [− 17.78 to − 4.87]	*< .001*	− 17.04 [− 24.05 to − 10.04]	*< .001*
End RT/CRT	3 months	9.37 [5.78–12.95]	*< .001*	20.87 [14.01–27.73]	*< .001*	23.76 [16.24–31.29]	* < .001*
OA75−							
Baseline	End RT/CRT	− 28.41 [− 32.17 to − 24.65]	*< .001*	− 46.27 [− 53.44 to − 39.09]	*< .001*	− 46.24 [− 53.90 to − 38.58]	* < .001*
Baseline	3 months	− 10.52 [− 14.36 to − 6.67]	*< .001*	− 14.01 [− 21.09 to − 6.39]	*< .001*	− 22.94 [− 30.60 to − 15.28]	*< .001*
End RT/CRT	3 months	17.89 [13.60–22.19]	*< .001*	32.26 [24.29–40.23]	*< .001*	23.30 [14.66–31.95]	* < .001*

Numbers in italic are significant

*Negative estimates indicate decreases

**Fig. 3:**
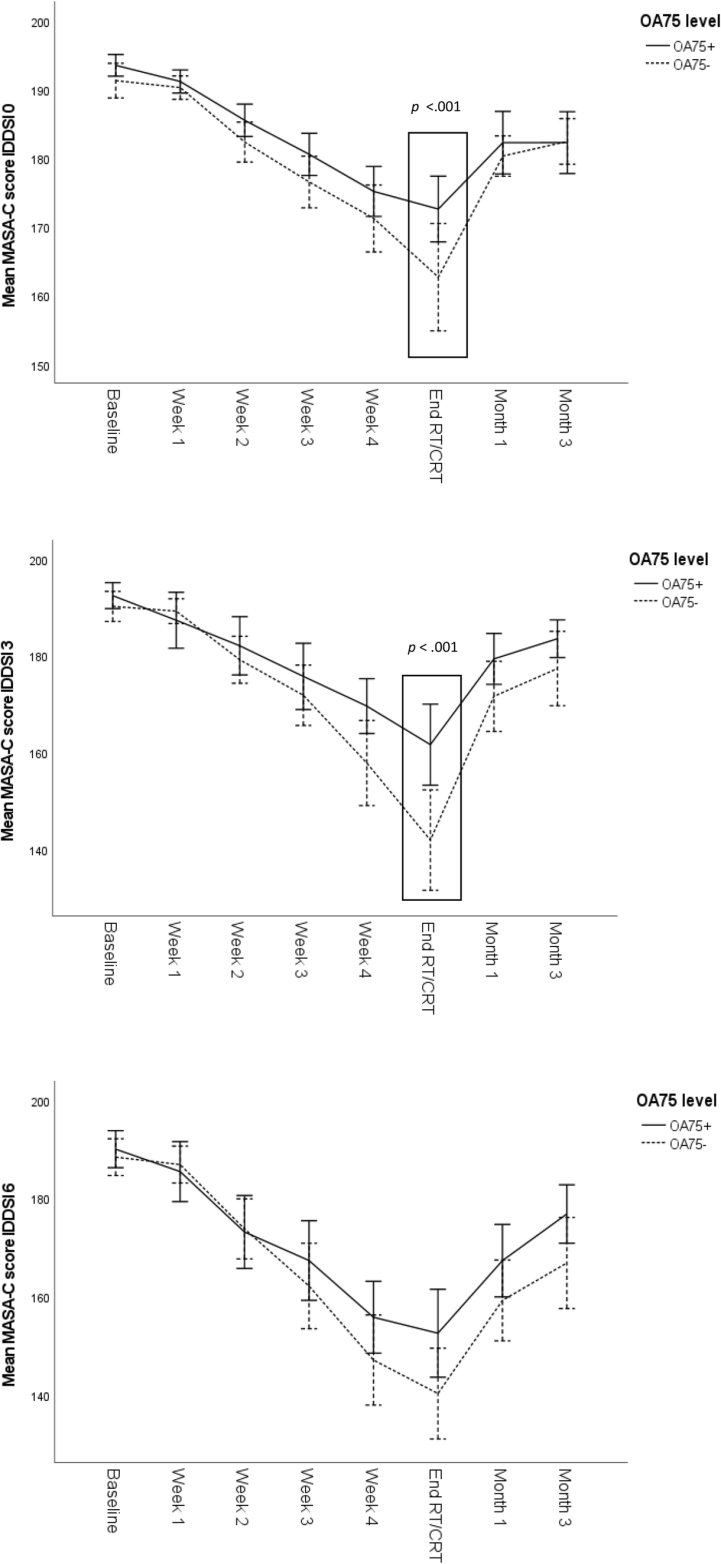
MASA-C scores through time by OA75 level, error bars: 95% CI

Post hoc analyses with Bonferroni–Holm correction show significant differences in MASA-C scores between adherence groups (OA75+ and OA75−) for IDDSI 0 and IDDSI 3 at the end of RT/CRT (*p* < 0.001). No significant differences were found for IDDSI 6. Figure 3 shows MASA-C scores through time by OA75 level with significant post hoc results indicated by means of a rectangle.

### Muscle Strength

Linear mixed effects model with time, group, and group by time interaction shows a significant interaction effect on percentage of MIP_shm_ gain (*F*_14–627_ = 5.258, *p* = 0.038). When correcting this model for adherence, with adherence as an ordinal variable (OA75+ vs. OA75−), this interaction effect is no longer observed, from which we infer that adherence, rather than group, affects muscle strength. Since group by time interaction is no longer significant, this variable was removed in our model.

In the linear mixed effects model with group, time, adherence, and adherence by time interaction, significant time effects are observed for MIP_a_ (*F*_7–604_ = 4.794, *p* < 0.001), MIP_p_ (*F*_7–575_ = 3.487, *p* = 0.001), Pswal_a_ (*F*_7–569_ = 2.858, *p* = 0.006), Pswal_p_ (*F*_7–528_ = 5.603, *p* < 0.001), and MIP_shm_ (*F*_7–575_ = 4.362, *p* < 0.001) with an increase in percentage of muscle strength gain for all measurements.

Significant effects of adherence are observed for MIP_a_ (*F*_1–113_ = 10.909, *p* = 0.001) and MIP_p_ (*F*_1–112_ = 8.992, *p* = 0.003) and adherence by time interaction is significant for MIP_a_ (F_7–603_ = 5.509, *p* < 0.001) and MIP_p_ (*F*_7–575_ = 2.221, *p* = 0.009).

**Fig. 4:**
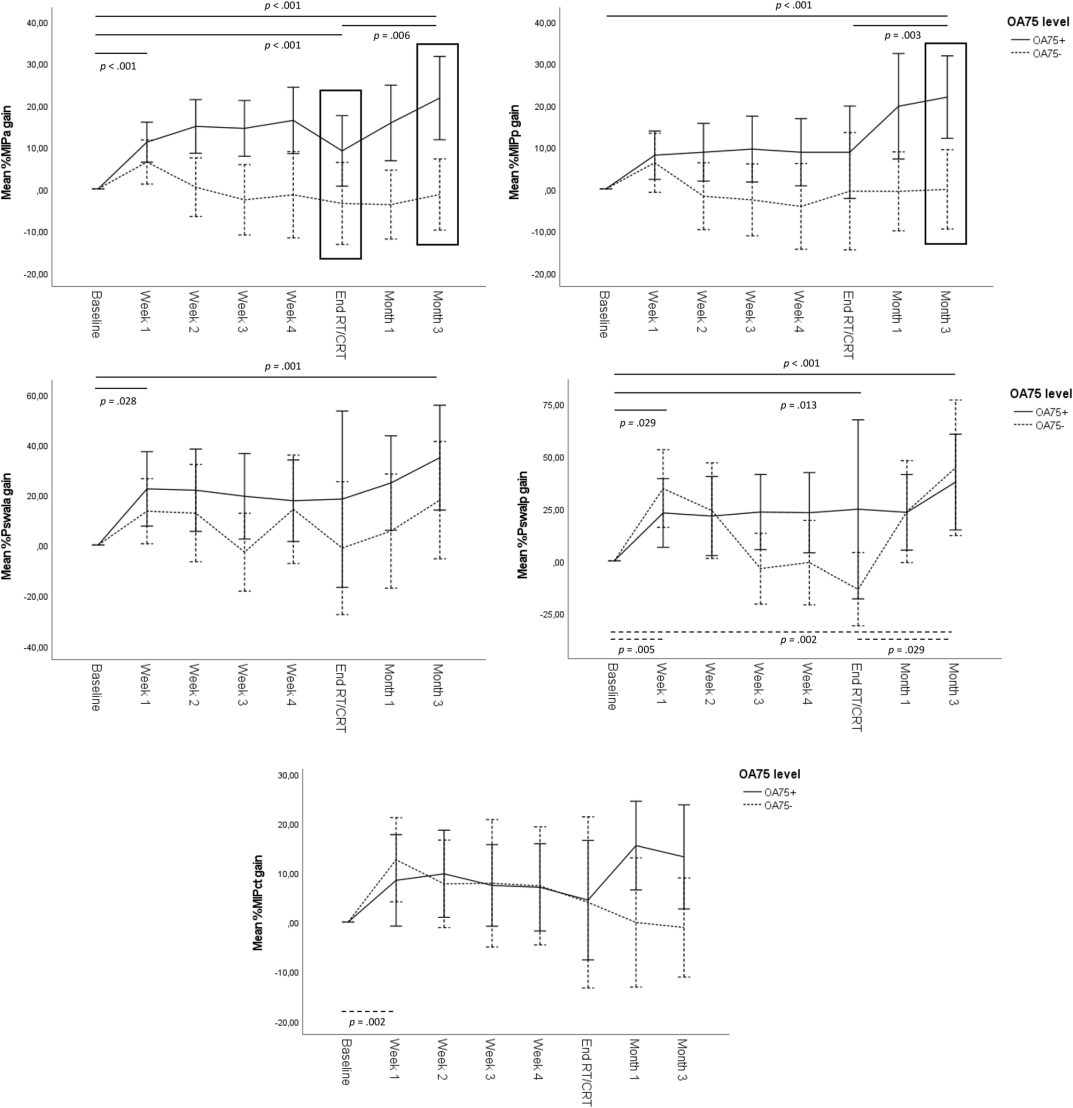
Percentages muscle strength gain through time by OA75 levels with significant post hoc results after Bonferroni–Holm correction, error bars: 95% CI

Post hoc analyses with Bonferroni–Holm correction for time are shown in Table 3; Fig. 4 shows the percentages of muscle strength gain through time by OA75 levels with significant post hoc results.

**Table 3:** Results of post hoc tests with Bonferroni–Holm correction for the evolution of muscle strength gain through time, depending on group

		MIP_a_		MIP_p_		Pswal_a_		Pswal_p_			MIP_shm_	
Difference in time-point												
		Estimate* (95% CI)	*p*	Estimate (95% CI)	*p*	Estimate (95% CI)	*p*	Estimate (95% CI)	*p*		Estimate (95% CI)	*p*
OA75+												
Baseline	Week 1	11.15 [5.95–16.34]	< *.001*	8.04 [1.60–14.47]	.087	22.34 [7.16–37.52]	*.028*	22.49 [6.13–38.86]	*.029*		8.44 [2.06–14.82]	.067
Baseline	End RT/CRT	10.08 [4.21–15.94]	< *.001*	5.05 [− 2.40–12.49]	.734	19.35 [2.10–36.61]	.168	30.75 [10.78–50.72]	*.013*		1.52 [− 5.93–8.97]	1
Baseline	3 months	20.75 [14.84–26.67]	< *.001*	19.69 [12.31–27.08]	< *.001*	33.36 [15.96–50.76]	*.001*	42.08 [23.23–60.92]	< *.001*		9.04 [1.71–16.36]	.094
End RT/CRT	3 months	10.68 [4.22–17.14]	*.006*	14.65 [6.44–22.85]	*.003*	14.01 [− 5.12–33.13]	.532	11.33 [− 10.64–33.30]	.623		7.52 [− .70–15.73]	.364
OA75−												
Baseline	Week 1	7.23 [1.46–13.01]	.057	5.93 [− 1.47–13.34]	.581	13.34 [− 4.19–30.87]	.532	32.92 [13.70–52.14]		*.005*	13.44 [6.21–20.66]	*.002*
Baseline	End RT/CRT	− 1.52 [− 9.27–6.22]	1	− 4.25 [− 14.72–6.22]	.850	− .12 [− 24.82–24.58]	.992	− 1.52 [− 31.21–28.17]		.920	− 2.34 [− 12.98–8.30]	1
Baseline	3 months	1.06 [− 6.00–8.12]	1	2.42 [− 6.50–11.33]	.850	21.13 [− .23–42.49]	.262	43.63 [19.85–67.42]		*.002*	− 1.30 [− 10.14–7.55]	1
End RT/CRT	3 months	2.56 [− 6.17–11.34]	1	6.67 [− 5.04–18.38]	.792	21.25 [− 6.49–48.99]	*.*532	45.15 [12.16–78.15]		*.029*	1.05 [− 10.70–12.79]	1

*MIP*_*a*_ anterior maximal isometric pressure, *MIP*_*p*_ posterior maximal isometric pressure, *Pswal*_*a*_ anterior tongue strength during swallowing, *Pswal*_*p*_ posterior tongue strength during swallowing, *MIP*_*shm*_ maximal strength of the suprahyoid muscles

Numbers in italic are significant

*Negative estimates indicate decreases

Post hoc analyses with Bonferroni–Holm correction show significant differences in percentage of MIP_a_ gain between adherence groups (OA75+ and OA75−) at the end of RT/CRT (*p* = 0.029) and at 3 months post-RT/CRT (*p* < 0.001). For MIP_p_, significant differences between adherence groups were observed at 3 months post-RT/CRT (*p* = 0.007). Figure 4 shows these significant post hoc results by means of a rectangle.

The sensitivity analysis for the missing adherence levels confirmed our results.

## Discussion

This multicenter randomized controlled trial investigated the effect of service-delivery mode (paper, app, and therapist supported) of prophylactic swallowing exercises (PSE) in HNC patients on swallowing function and muscle strength during and after RT/CRT treatment. No significant effects of service-delivery mode were found. This is consistent with the study by Wall et al., in which no significant effects of service-delivery mode were observed for all swallowing and nutrition-related outcomes [25]. Additionally in our study, participants were divided according to their overall adherence level, independently of their assigned service-delivery mode. Results showed significantly better swallowing function and muscle strength gain in patients practicing ≥ 75% (OA75+) of the prescribed exercises compared to patients practicing < 75% (OA75−).

To our knowledge, there are no other studies that investigated the effect of PSE on instrumentally measured muscle strength during and after RT/CRT. Carroll and colleagues suggested, however, that in patients who performed PSE during RT/CRT, tongue base muscle mass may be better preserved than in patients who did not, due to less atrophy [26]. Furthermore, the randomized controlled trial of Carnaby-Mann et al. observed less structural deterioration in muscle composition in patients performing PSE [2]. Both findings are in line with the overall positive effect of PSE on muscle strength in the PRESTO trial.

It is, however, remarkable that our patients were able to increase their tongue and suprahyoid muscles strength, despite the acute toxicities. To our knowledge, this is the first study demonstrating an actual and significant increase in muscle strength during RT/CRT by means of strengthening exercises. Hereby, it is important to notice that the degree of adherence matters: tongue strength increases significantly in the OA75+ group compared to the OA75− group, where the strength is more likely to remain stable or decrease during RT/CRT. A high intensity of exercise, translated in PRESTO as 5 days a week combined with a high number of repetitions per session, and the use of devices that provide biofeedback is key to show positive effects on muscle strength. The importance of the principles of motor learning and strength training is clearly illustrated here [20].

Despite the shown reliability of all strength measures used [24, 27], remarkable increases were demonstrated for all muscle strength measures between baseline and week 1 of RT/CRT. To improve the precision of the assessment and to exclude learning curve effects, both Adams and Kraaijenga et al. suggest the use of a familiarization session before baseline measurements [24, 27]. Current study did not use a familiarization session before the effective strength measurements. Although, since the large increases in strength, it is our hypothesis that familiarization with the devices cannot be the only explanation of this remarkable phenomenon. A probable explanation for this rapid and significant improvement may be found in the physiology of strength training. During the initial phase of strength training, adaptations occur in the way the nervous system activates the muscles. When an individual starts performing strength training, a learning process occurs that allows for the correct recruitment and firing rate of the relevant motor units, as well as de-activation of antagonistic muscles. This also occurs in tongue muscles: learning improves performance and induces plasticity in corticomotor pathways [28]. Changes in the coordination of motor unit recruitment occur as well as changes in the learning how to improve this recruitment and thus improve muscle activation during a specific strength task. In this way, the learning effect causes an increase in strength, without necessarily achieving an increase in muscle mass. In a later phase of strength training, structural changes in the muscles themselves will occur: growth in muscle size and changes in muscle composition follow the improvement in strength [20, 29, 30].

After week 1, the OA75+ group was able to maintain the strength improvement during and until the end of RT/CRT. This plateau effect was not present in the OA75− group. Van den Steen et al. evaluated the feasibility of tongue strength measures during RT/CRT in HNC patients, not performing PSE. Consistent with our results in the OA75− group, a decrease in MIP_a_ and MIP_p_ was observed [31].

After RT/CRT, no detraining effects were found for any of the five strength measurements. Moreover, between the end of RT/CRT and 3 months post-RT/CRT, a significant increase was found for both anterior and posterior tongue strength in the OA75+ group. Possible explanations for this continuous increase could be the decrease in acute toxicity (mucositis, pain) or an improvement in oral intake. However, since the increase in tongue strength after RT/CRT was not found in the OA75− group, it can be suggested that an effective improvement in muscle strength in the OA75+ group occurred.

Despite the increase in muscle strength and its transference to swallowing strength, a significant decrease in swallowing function during RT/CRT was still observed. Between baseline and the end of RT/CRT, a strong deterioration was seen, followed by a recovery, however not to baseline levels. These results are consistent with other studies in which intensive preventive swallowing therapy is applied [2, 3, 7, 8, 25]. Van der Molen et al., for example, described a significant decrease in oral intake during RT/CRT in patients performing PSE. However, previous research also showed that patients performing PSE showed beneficial effects on post-treatment swallowing function compared to control groups, not performing PSE [1, 2, 26]. The current study did not include a control group, but the results show that higher adherence to PSE results in less deterioration of the swallowing function. This is in line with the results of previous research [6, 13]. Duarte et al. [6], for example, evaluated patients receiving PSE during RT/CRT and showed that swallowing function was better preserved at the end of RT/CRT in patients adherent to PSE.

Since we know that there are significant differences in adherence among the three service-delivery modes, we assume that the differences in swallowing function and muscle strength between groups were mainly due to the differences in adherence.

Our study is, however, not without limitations. A rather short longitudinal follow-up period prevents us from making any conclusions on the long term. Since chronic radiation-associated dysphagia is common and highly impacting on health-related QoL in HNC survivors, this prospective study should ideally be conducted up to a year or even several years after RT/CRT. Examining the effects of PSE on swallowing function > 1 year and up to 5–10 years after RT/CRT, is subject for future research. The lack of data concerning muscle composition prevented us from making statements about actual muscle changes. Although it is an assumption, we were unable to conclude with certainty that muscle hypertrophy occurred. Moreover, no objective measures, e.g., flexible endoscopic swallowing examination or videofluoroscopy, were conducted. It is possible that when PSE is performed, less residue occurs or that specific swallowing characteristics, such as epiglottic inversion or tongue base retraction, are better preserved. It would also be interesting to compare the more detailed OA levels based on Wall et al. (≥ 75%, 50–75%, 25–50%, ≤ 25%) [14]; however, our groups were too small and the associated statistical models too weak. Lastly, a familiarization session with the devices is something to take into account in future studies in order to increase the accuracy of the measurements.

Next to these limitations, questions can arise concerning the study design, and in particular the duration of the PSE program. The choice to limit of the duration of the program to the first 4 weeks of radiotherapy involved a huge amount of thoughts and brainstorming. The entire research team provided input, and the decision was finally based on different reasons: on the one hand, previous studies within our own research team showed that adherence decreases toward the end of radiotherapy. On the other hand, it is well known that acute toxicity peaks in the 5th week of irradiation, and the aim of our trial was to build up functional reserve in the period that is least burdensome for the patients [8]. In addition, we wanted to give the patients a perspective in order to keep the adherence rates as high as possible. Longer practice might have a greater impact on outcomes, but the aim of current study was to determine whether intensive practice during the first 4 weeks of radiotherapy could lead to significant differences in outcomes, which was demonstrated based on our results. Moreover, research executed in subjects without dysphagia shows already significant increases in tongue strength and tongue strength during swallowing after 4 weeks of intensive rehabilitation [32, 33].

In addition, questions concerning the access and cost of this large number of IOPIs to lend out to patients, can also arise. In the hospital setting and in private practice in Belgium, however, the IOPI device is becoming more and more established. It is the most simple clinical instrument to train the main swallowing muscles with a lot of advantages like visual and tactile feedback, and it seems financially feasible.

As mentioned above, previous literature already showed that patients with HNC, following a prophylactic swallowing exercise (PSE) program, have better swallowing function when they adhere to this program compared to patients who are not adherent [6, 13]. Wall et al. observed differences in adherence rates depending on service-delivery mode. However, in this study, adherence rates were found to be moderate to low [14]. Since PRESTO showed how to keep the adherence rates high, we wanted to investigate the effects of PRESTO on swallowing function and muscle strength. Therefore, this multicenter randomized controlled trial fills the gap of investigating the impact of PSE on different outcome measures while achieving high overall adherence. Furthermore, by concluding the importance of minimum 75% overall adherence, the SLP has a clinical recommendation to inform patients concretely about the conditions and expectations from this prophylactic program.

Further steps within our PRESTO trial are to investigate the influencing factors for (non)adherence. Analysis of patient-related factors, e.g., personality, general condition, and fatigue, will be done in follow-up studies.

In conclusion, our randomized controlled trial found no effects of service-delivery mode of PSE on swallowing function or muscle strength. However, significant effects were found with respect to the patients’ overall adherence level. Patients practicing more than 75% of the prescribed exercises showed significant better results in swallowing function and muscle strength. It can be concluded that a high level of exercise repetitions is essential to achieve benefits of PSE during RT/CRT.

## Data Availability

The datasets generated during the current study are not publicly available since they contain patient data and the Informed Consent does not include sharing data publicly. They are available from the corresponding author upon reasonable request. All clinical record forms are collected and managed by using REDCap (Research Electronic Data Capture) electronic data capture tools hosted at Ghent University Hospital [1].
